# Correlation of vascular enhancement on MRI and clinical manifestations in patients with GCA

**DOI:** 10.1093/rap/rkag060

**Published:** 2026-07-03

**Authors:** Nitin S Rai, Azin Khosavirad, Lawrence Mbuagbaw, Rennie L Rhee, Ryan Rebello, Nader Khalidi, Mats Junek

**Affiliations:** Department of Medicine, McMaster University, Hamilton, ON, Canada; Department of Health Research Methods, Evidence, and Impact (HEI), McMaster University, Hamilton, ON, Canada; Department of Health Research Methods, Evidence, and Impact (HEI), McMaster University, Hamilton, ON, Canada; Department of Anesthesia, McMaster University, Hamilton, ON, Canada; Department of Pediatrics, McMaster University, Hamilton, ON, Canada; Research Methodology Centre, The Research Institute of St Joe’s, St. Joseph’s Healthcare, Hamilton, ON, Canada; Centre for Development of Best Practices in Health (CDBPH), Yaoundé Central Hospital, Yaoundé, Cameroon; Division of Epidemiology and Biostatistics, Department of Global Health, Stellenbosch University, Cape Town, South Africa; Mary Heersink School of Global Health & Social Medicine, McMaster University, Hamilton, ON, Canada; Division of Rheumatology, University of Pennsylvania, Philadelphia, PA, USA; Department of Diagnostic Imaging, McMaster University, Hamilton, ON, Canada; Department of Anesthesia, McMaster University, Hamilton, ON, Canada; Division of Rheumatology, Department of Medicine, McMaster University, Hamilton, ON, Canada; Department of Health Research Methods, Evidence, and Impact (HEI), McMaster University, Hamilton, ON, Canada; Department of Anesthesia, McMaster University, Hamilton, ON, Canada; Division of Rheumatology, Department of Medicine, McMaster University, Hamilton, ON, Canada

**Keywords:** GCA, MRI, cranial vessel, temporal artery

## Abstract

**Objectives:**

GCA has diverse cranial manifestations, and cranial vessel MRI (cvMRI) can assess multiple arteries simultaneously. We aimed to determine whether vessel-specific enhancement on cvMRI correlates with clinical manifestations and diagnosis in GCA.

**Methods:**

We conducted a retrospective cohort study of patients who underwent cvMRI for suspected GCA at a single academic centre. We assessed associations between vessel wall enhancement on cvMRI and anatomically expected GCA symptoms. We used univariate and multivariable logistic regression adjusting for age, sex and glucocorticoid exposure to assess the relationship between vessel abnormalities and a diagnosis of GCA.

**Results:**

Among 439 patients, 153 (34.9%) were diagnosed with GCA. Median time (quartile 1 [Q1]–quartile 3 [Q3]) from glucocorticoid initiation to cvMRI was 11.5 days (Q1–Q3: 5.8–207.2). Maxillary and ophthalmic artery involvement were specific but insensitive for jaw claudication and vision loss, respectively (specificity 87.1%, 95% CI, 80.3–93.9 and 72.6%, 95% CI, 61.5–83.7; sensitivity 40.0%, 95% CI, 26.5–51.4 and 43.3%, 95% CI, 33.1–53.6, respectively). Headache showed no association with specific vessels. Any arterial enhancement was associated with GCA in univariate analysis, but only temporal artery involvement was predictive in multivariable analysis (odds ratio [OR] 5.20, 95% CI, 1.65–16.88), particularly when bilateral (OR 9.82, 95% CI, 2.82–38.32).

**Conclusion:**

Temporal artery enhancement remains the strongest imaging correlate for a diagnosis of GCA. Vessel-specific enhancement on cvMRI correlates with vision loss and jaw claudication but not headache. cvMRI may aid diagnosis in diagnostically uncertain cases, even after glucocorticoid initiation.

Key messages

- The temporal artery is the most reliable imaging marker for GCA.
- Maxillary and ophthalmic artery enhancement is specific for their relevant symptoms but lack sensitivity.
- MRI is particularly useful in patients with isolated vision loss, jaw claudication or atypical cases, where ultrasound may be inconclusive.

## Introduction

GCA is the most common large vessel vasculitis and typically presents in females aged 50 years or older [1]. Diagnosing GCA remains challenging for many patients as symptoms can be non-specific and variable; the classic presentation of headaches, jaw claudication and scalp tenderness are seen in only 30–40% of patients [2]. Temporal artery biopsy (TAB), previously considered the gold standard test, has low sensitivity [3, 4]. The use of imaging such as temporal artery ultrasound (TA-US) and cranial vessel wall MRI (cvMRI) has revolutionized how GCA is diagnosed over the last decade [5, 6]. This use of imaging has facilitated the diagnosis of GCA, development of GCA fast track clinics and earlier treatment, potentially reducing vision loss associated with the disease [7]. The role of cvMRI in the diagnosis of GCA is still being explored; while it may be more logistically challenging to perform, it can evaluate multiple cranial vessels and other structures simultaneously [8–10].

The use of imaging modalities has also allowed for a more in-depth understanding of GCA. Vertebral artery involvement is increasingly recognized as a potential driver of the higher frequency of posterior strokes in GCA; multiple orbital manifestations of GCA may allow for earlier recognition of individuals at risk of vision loss who may benefit from more aggressive therapy [11]. These findings also call into question whether temporal artery involvement is the hallmark of cranial GCA or simply the most accessible vessel for biopsy and TA-US. The use of cvMRI allows for systematic evaluation of all cranial arteries in GCA; data published by Rhee et al. [12] have suggested that the occipital and maxillary artery may be involved as frequently as the temporal artery and ophthalmic artery. Such data will also allow for a more fulsome exploration of symptom-vessel correlations in GCA.

We assessed whether there is a correlation between clinical signs and/or symptoms of GCA and anatomically correlated blood vessels in GCA using cvMRI. We also assessed the extent to which abnormal findings in each vessel may contribute to a diagnosis of GCA.

## Methods

### Study design

We conducted a retrospective analysis using the McMaster GCA database combined with secondary analysis of de-identified data from the study by Rhéaume et al. [5]. The study received approval from HiREB (approval 17429). The McMaster GCA database includes individuals who are being considered for a possible diagnosis of GCA and consented to participate in a longitudinal, prospective database that has had ongoing consent and participation from July 2015 onward. Participants in the study of Rhéaume et al. were 171 individuals being similarly assessed for a possible diagnosis of GCA and underwent both TAB and cvMRI to assess for a possible GCA diagnosis from February 2007 to March 2014. No individuals participated in both the database and the study of Rhéaume et al.

### Inclusion and exclusion criteria

Participants included in this study were adults aged 50 years or older who were suspected of having GCA and had undergone cvMRI. Patients without results of cvMRI, where their final diagnosis is unknown or lack of follow-up information were excluded from the analysis.

### Outcomes and data collection

Outcomes included a diagnosis of GCA and signs and/or symptoms of GCA including typical headache, jaw claudication and/or vision loss. Typical headache was defined as a temporal headache onset within the last 6 weeks not responsive to conventional analgesia. All outcomes were assessed using standardized definitions that were extracted from routine clinical documentation from visits with individuals assessed by their treating clinician. Participants were considered to have GCA if the final clinical diagnosis was confirmed at a minimum of 6 months’ follow-up after the initial visit. Vision loss was defined as a permanent decrease in visual acuity associated with onset of GCA with an ocular syndrome compatible with a diagnosis of GCA as assessed by ophthalmology. Vision changes were defined as visual symptoms that completely resolved or were not associated with a decrease in visual acuity or irreversible ocular changes based on ophthalmologic evaluation.

Data from each cohort used for this analysis included: demographics, past cardiovascular history, signs or symptoms at presentation for evaluation of GCA, investigation results (including cvMRI, CRP and erythrocyte sedimentation rate), final diagnosis, treatment with glucocorticoids and treatment-imaging interval and outcomes. Information concerning cardiovascular risk factors was only available from the GCA database. Vessels routinely assessed by cvMRI included the common temporal artery, its frontal and parietal branches and the maxillary, ophthalmic, occipital, vertebral and internal carotid arteries. Vessel abnormality was determined based on the findings of the reporting radiologist, using a consensus definition in which an abnormal vessel was defined as having grade two or higher abnormalities using the system of Bley et al. [13].

### Statistical analysis

We summarized the cohort and cvMRI findings separately using frequency, mean, S.D., median and quartiles as appropriate. We evaluated the diagnostic accuracy of cvMRI in the cohort using sensitivity, specificity, positive predictive value and negative predictive value. A Kappa matrix was used to analyse agreement between signs and vessel involvement, and then to assess agreement between the laterality of symptoms and vessel abnormalities on imaging. In assessing sides, the unit of measure was the left and right side of the individual rather than the individual. Univariate then multivariate logistic regression models were used to assess the relationship between final clinical GCA diagnosis and each blood vessel territory affected, controlling for age and sex and reported using odds ratios (ORs) and their corresponding 95% CIs. Multivariable analysis was repeated both including and excluding the time interval between glucocorticoid treatment initiation and imaging due to incomplete reporting of this finding as well as considering any vessel positivity versus the number of times each vessel was positive (i.e. if there was unilateral or bilateral temporal positivity). Multivariable analysis was repeated for the outcomes of typical headache, jaw claudication, any vision changes and vision loss. This study adhered to STROBE guidelines for observational research [14].

## Results

**Figure 1:**
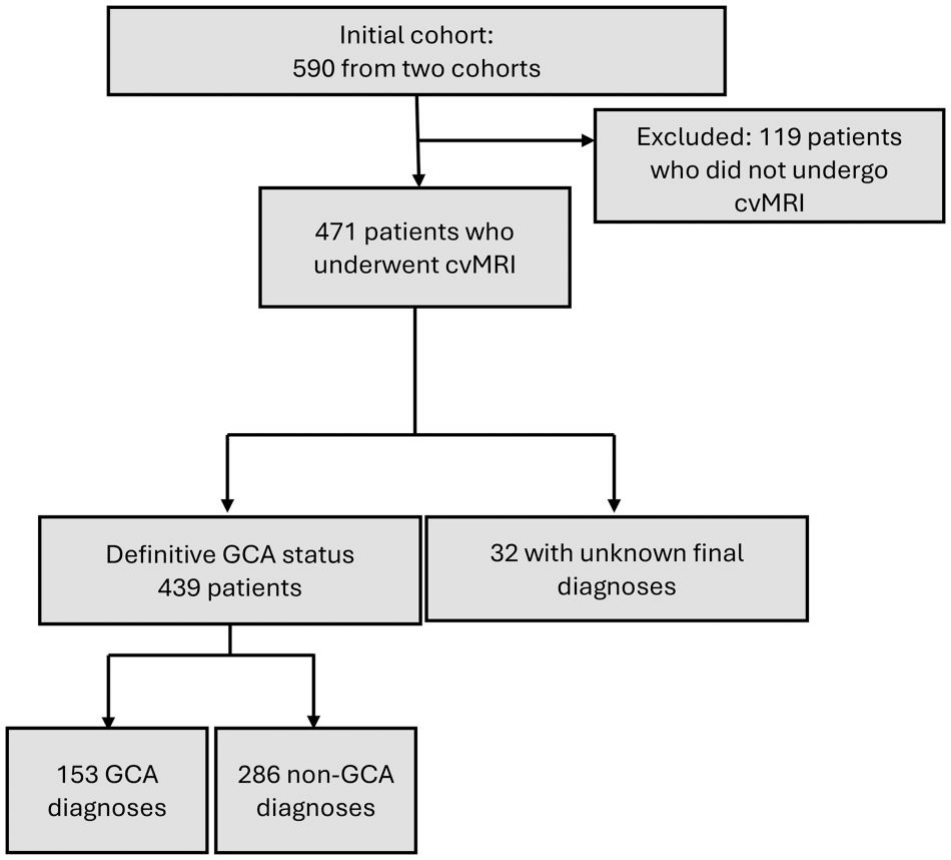
Patient flow diagram

There were 590 individuals in the two cohorts; 119 who did not undergo cvMRI were excluded and 32 patients who underwent cvMRI without a recorded diagnosis were also excluded (Fig. 1). Complete data were available for 439 patients who both underwent cvMRI and had a known GCA diagnostic status. Of these, 153 (34.9%) were diagnosed with GCA and 104 (68.0%) of those with GCA had a positive cvMRI (Table 1). The sensitivity of cvMRI for a diagnosis of GCA was 67.97% (95% CI, 60.58–75.36) and specificity was 88.46% (84.75–92.16). TAB was performed in 234 (53.3%) and was positive in 44 (28.8%) of those with GCA. TA-US was only routinely introduced into use in ∼2020; 94 (21.4%) individuals underwent TA-US and 14 (3.2%) were positive. The interval between glucocorticoid administration and imaging was available for 350 of the 439 (79.72%) individuals who underwent cvMRI. In individuals diagnosed with GCA, those with an abnormal cvMRI had a median of 12 (Q1–Q3: 6–208) days of glucocorticoid exposure before imaging and those with a normal cvMRI had median of 15 (Q1–Q3: 7–23) days of exposure.

**Table 1:** Baseline characteristics of the study participants undergoing cvMRI (*n* = 439).

Characteristic	No GCA diagnosis (*N* = 286)	GCA diagnosis (*N* = 153)	Total (*N* = 439)
Age (years) (median [Q1, Q3])	67.0 (59.0, 74.0)	74.0 (66.0, 79.0)	70.0 (61.0, 77.0)
Sex (female), *n* (%)	200 (69.9%)	107 (69.9%)	307 (69.9%)
Comorbidities			
Hypertension (%)	92 (32.2%)*	44 (28.8%)*	136 (31.0%)*
Dyslipidaemia (%)	54 (18.9%)	28 (18.3%)	82 (18.7%)
Diabetes mellitus (%)	29 (10.1)*	16 (10.5%)	45 (10.3%)
Peripheral vascular disease (%)	2 (0.7%)	1 (0.7%)	3 (0.7%)
Smoking (ever smoked) (%)	69 (24.1%)*	33 (21.6%)*	102 (23.2%)*
Presenting symptoms			
Any headache, *n* (%)	249 (87.1%)	118 (77.1%)	367 (83.6%)
Typical headache, *n* (%)	115 (40.2%)	38 (24.8%)	153 (34.9%)
Scalp tenderness, *n* (%)	108 (37.8%)	66 (43.4%)	174 (39.6%)
Temporal artery abnormality, *n* (%)	102 (35.7%)	57 (37.3%)	159 (36.2%)
Jaw claudication, *n* (%)	59 (20.6%)	59 (38.6%)	118 (26.9%)
Any vision change, *n* (%)	139 (48.6%)	90 (58.8%)	229 (52.2%)
Vision loss, *n* (%)	33 (11.5%)	37 (24.2%)	70 (15.9%)
Other vision changes, *n* (%)	121 (42.3%)	78 (51.0%)	199 (45.3%)
Investigations			
CRP (mg/dl) (median [Q1, Q3])	5.00 [1.6, 16.0]	19.4 [5.25, 53]	7.60 [2.10, 26.98]
ESR (mm/h) (median [Q1, Q3])	22.0 [9.0, 42.2]	41 [22, 70]	29.0 [11.5, 50.0]
Underwent TAB, *n* (%)	115 (40.2%)	119 (77.8%)	234 (53.3%)
TAB positive (%)	0 (0.0%)	44 (28.8%)	44 (10.0%)
Underwent TA-US (%)	64 (22.4%)	30 (19.6%)	94 (21.4%)
TA-US positive (%)	5 (1.7%)	9 (5.9%)	14 (3.2%)
cvMRI positive, *n* (%)	33 (11.5%)	104 (68.0%)	137 (31.2%)

Data concerning comorbidities was only available from the McMaster GCA database and is thus presented as frequencies. Variables marked with an asterisk (*) had >20% missing data; only percentages (%) are reported. TAB: temporal artery biopsy; TA-US: temporal artery ultrasound; cvMRI: cranial vessel wall MRI.

The most frequently positive vessel was the temporal artery in 92 individuals (60.1% of those with GCA) and the least frequently positive vessel was the vertebral artery in 4 (1.7% of those with GCA) (Table S1, available at *Rheumatology Advances in Practice* Online). Out of the 67 individuals (43.8%) with an abnormal occipital artery, only four had isolated occipital artery abnormalities without concurrent temporal or vertebral artery involvement. There were no individuals with isolated vertebral artery involvement. When assessing the relationship between glucocorticoid exposure and cvMRI positivity, cumulative duration of glucocorticoids was not associated with a difference (OR 1.00, 95% CI, 0.99–1.004). It was noted that there was a rightward skew, and when those with 30 days or longer of glucocorticoid exposure were excluded (*n* = 75, 20.0% of those with glucocorticoid exposure data), we found that each day of glucocorticoid exposure was associated with decreased odds of cvMRI positivity, OR 0.95 (95% CI, 0.91–0.99) after adjusting for age and sex. This effect was consistent when assessing across temporal, ophthalmic and occipital arteries individually (ORs ranging 0.94–0.95, 95% CIs ranging 0.91–0.99).

Maxillary and ophthalmic arteries were specific for the corresponding symptoms of jaw claudication and vision changes/loss (Specificity 87.1% [95% CI, 80.3–93.9] and 72.6% [95% CI, 61.5–83.7], respectively) but had low sensitivity (40.0% [26.5–51.4] and 43.3% [33.1–53.6], respectively) (Table 2). Temporal and/or occipital findings were of low specificity for either typical or atypical headache. Among the two individuals with a positive TAB but negative cvMRI, the mean glucocorticoid exposure prior to imaging was 10.5 days. When considering the laterality of symptoms and changes on cvMRI, it was found that there were similar frequencies of individuals with symptoms both concordant and discordant with the side of cvMRI changes (Table S2, available at *Rheumatology Advances in Practice* Online).

**Table 2:** Sensitivity and specificity of blood vessels for their expected anatomic correlate.

Affected blood vessel	Expected symptom	Sensitivity	Specificity	Positive predictive value	Negative predictive value
Temporal artery	Temporal artery change/scalp tenderness	57.95 (47.64, 68.26)	35.38 (23.76, 47.00)	55.83 (44.72, 64.95)	38.34 (26.03, 50.63)
Temporal artery	Typical headache	71.05 (56.63, 85.47)	35.13 (19.75, 50.51)	52.94 (39.24, 66.64)	54.16 (34.23, 74.10)
Occipital artery	Atypical headache	37.50 (13.78, 61.22)	49.15 (36.39, 61.90)	16.67 (4.49, 28.84)	74.36 (60.65, 88.06)
Temporal and/or occipital	Any headache (typical or atypical)	58.47 (49.58, 67.36)	20.00 (6.75, 33.25)	71.13 (62.11, 80.15)	12.50 (3.84, 21.16)
Maxillary/facial artery	Jaw claudication	39.98 (26.53, 51.42)	87.09 (80.28, 93.91)	65.71 (49.98, 81.43)	69.23 (60.86, 77.59)
Ophthalmic artery	Vision loss/vision changes	43.34 (33.09, 53.57)	72.58 (61.47, 83.68)	69.64 (57.59, 81.68)	46.87 (36.89, 56.85)

Results are presented as the value and its 95% CI.

In assessing the association between arterial abnormalities on cvMRI and a diagnosis of GCA, internal carotid artery abnormalities demonstrated the highest unadjusted OR (OR 27.41, 95% CI, 5.02–510.21), followed by the superficial temporal artery (16.12, 9.69–27.67) and ophthalmic artery (13.32, 7.07–27.05) (Table 3). However, in multivariable analysis, only superficial temporal artery abnormalities remained independently predictive of a GCA diagnosis (OR 5.20, 1.65, 16.88), the point estimate was higher when there was bilateral temporal artery involvement (9.82, 2.82–38.32). Bilateral maxillary artery involvement also had a high point estimate but a wide CI (7.03, 0.70–180.38). The analysis was unchanged after repeating without glucocorticoid exposure duration (data not shown).

**Table 3:** Logistic regression for vessel involvement on cvMRI and GCA diagnosis.

Affected blood vessel	Unadjusted odds ratio (95% CI) (*univariate analysis)*	Adjusted odds ratio (95% CI) *(multivariable analysis)*	Stratified odds ratio (95% CI) for bilateral vs unilateral involvement
Temporal artery	16.12 (9.69, 27.67)	5.20 (1.65, 16.88)	Unilateral: 1.62 (0.24, 8.61) Bilateral: 9.82 (2.82, 38.32)
Maxillary artery	21.09 (8.20, 71.75)	1.24 (0.22, 9.67)	Unilateral: 0.06 (0.00, 0.86) Bilateral: 7.03 (0.70, 180.38)
Ophthalmic artery	13.32 (7.07, 27.05)	2.77 (0.67, 13.30)	Unilateral: 1.22 (0.13, 27.24) Bilateral: 2.18 (0.41, 12.40)
Occipital artery	12.01 (6.90, 21.87)	2.92 (0.79, 11.11)	Unilateral: 2.09 (0.18, 51.35) Bilateral: 2.28 (0.76, 15.25)
Internal carotid artery	27.41 (5.02, 510.21)	9.45 (0.37, 575.70)	N/A^a^
Vertebral artery	5.63 (1.08, 41.27)	6.41 (0.19, 420.00)	N/A^a^
Intracranial artery^b^	8.51 (2.80, 31.55)	0.17 (0.01, 3.49)	N/A^a^
Age	1.07 (1.05, 1.09)	1.01 (0.98, 1.05)	1.01 (0.97, 1.05)
Sex	1.00 (0.65, 1.54)	2.15 (0.92, 5.38)	2.87 (1.16, 8.00)
Days of glucocorticoid exposure before imaging		1.01 (1.00, 1.01)	1.00 (1.00, 1.00)

a Insufficient number of abnormal vessels.

b Includes middle cerebral artery (MCA), anterior cerebral artery (ACA) and posterior cerebral artery (PCA). cvMRI: cranial vessel wall MRI.

After analysing associations between symptoms and vessel involvement in Table 4, all 95% CIs included were wide and included one indicating low precision. Ophthalmic artery involvement demonstrated the highest OR for vision loss (OR 2.60, 95% CI, 0.81–8.65). Increasing age was associated with increased odds of vision loss (OR 1.03, 95% CI, 1.00–1.07).

**Table 4:** Logistic regression for symptoms and vessel involvement (either side) expressed as odds ratio and their 95% CIs.

Variable	Typical headache (OR; 95% CI)	Vision loss (OR; 95% CI)	Jaw claudication (OR; 95% CI)
Age	0.98 (0.96, 1.00)	1.03 (1.00, 1.07)	0.97 (0.94, 1.00)
Female sex	1.17 (0.70, 1.95)	0.59 (0.29, 1.22)	0.80 (0.45, 1.43)
Temporal artery	1.32 (0.60, 2.97)	0.42 (0.09, 1.42)	1.50 (0.58, 3.62)
Maxillary artery	1.22 (0.36, 4.58)	1.00 (0.21, 4.01)	1.85 (0.53, 6.40)
Ophthalmic artery	1.33 (0.52, 3.50)	2.60 (0.81, 8.65)	1.22 (0.45, 3.25)
Occipital artery	0.98 (0.37, 2.55)	2.43 (0.65, 10.61)	1.47 (0.54, 4.11)
Internal carotid artery	0.48 (0.11, 1.92)	0.69 (0.09, 3.66)	3.05 (0.77, 12.68)
Vertebral artery	0.17 (0.01, 1.33)	2.06 (0.23, 15.31)	0.68 (0.07, 4.68)

Abbreviation: OR: odds ratio.

## Discussion

We demonstrated that multiple cranial arteries including the temporal, maxillary, ophthalmic and occipital arteries may be involved in GCA, but the temporal artery continues to be the best predictor of a diagnosis after adjustment for abnormalities in other cranial vessels. We also confirmed the correlation between jaw claudication and vision changes in GCA and the arteries supplying the relevant anatomy. We also found that symptoms are specific but relatively insensitive for specific arterial involvement in a cohort where imaging frequently occurred after a median of 11 days of glucocorticoids exposure. These findings suggest that in patients presenting with isolated vision loss, jaw claudication or atypical symptoms, cvMRI may be particularly helpful, given its ability to assess the entire cranial vascular tree non-invasively.

We observed that involvement of any cranial artery on imaging was associated with a diagnosis of GCA. This reinforces the value of comprehensive vessel assessment using cvMRI and suggests that more limited assessment on TA-US may miss certain vessels. In adjusted models, only temporal artery enhancement remained independently predictive of GCA, consistent with its status as the most frequently positive artery and its anatomical role as the proximal stem of many anterior cranial branches. This finding supports its continued use as a primary imaging target in clinical assessment and aligns with prior studies showing that temporal artery abnormalities increase diagnostic confidence for GCA [8, 10, 15]. While limited by sample size, the high point estimate associated with bilateral abnormalities in any artery suggests that symmetric involvement, when present, should further raise diagnostic suspicion, particularly in patients with otherwise equivocal signs and/or symptoms. Delayed imaging following glucocorticoid initiation may also explain the low prevalence of isolated occipital artery involvement observed in our cohort, as it may preferentially reduce detection of less frequently involved or smaller vessels.

Our findings agree with those of Rhee et al. [12] which demonstrate that abnormalities of the ophthalmic artery are associated with vision loss. Multivariate regression did not, however, find this to be independently predictive of vision loss. Differences in MRI protocol, specifically the absence of dedicated orbital imaging in this study, and inability to assess optic nerve sheath may explain the lack of association with MRI and vision loss in this study. It also suggests the presence of an ophthalmic arterial abnormality may warrant escalation of therapy or closer observation of vision, even in the absence of other findings. Limited evidence suggests that ultrasound may also be helpful in identifying abnormal orbital structures in GCA, however, this only assesses the optic nerve sheath and may miss other structures that can be identified by cvMRI [16]. Similarly, jaw claudication has been associated with changes in the muscles of mastication as well as maxillary artery abnormalities, which may explain some of the low sensitivity of this finding. cvMRI also highlights other jaw structures and can illustrate other non-GCA pathologies that mimic a clinical diagnosis of GCA such as TMJ disorders [15, 17, 18]. The maxillary arteries, because of their location deep to the mandible, cannot be assessed with ultrasound. These findings further support the role of cvMRI as a valuable second-line modality in cases with high clinical suspicion for GCA and negative TA-US results [19].

In contrast, headaches did not show a consistent correlation with specific arterial involvement despite being the most frequently reported symptom. Prior literature suggests that headaches in GCA may be associated with temporal artery abnormalities, though this association is not consistently observed [20, 21]. This may reflect that headaches are a result of systemic inflammation or disease activity not well visualized on cvMRI such as the deep temporal artery, which has been reported on MRI in GCA [22] We also saw that there was no concordance between the side of symptoms and the side of cvMRI abnormalities indicating poor lateralization. Similar findings were seen by Maleszewski et al. [23] who demonstrated detectable arterial abnormalities in individuals who underwent serial TAB where the first one was done on the more symptomatic side. Collectively, these findings suggest that as many vessels as possible should be imaged to maximize diagnostic evidence of GCA. In addition, several cases of MRI positivity occurred in the absence of the corresponding symptom, suggesting subclinical involvement. While ultrasound can be used to assess extracranial vessels such as the subclavian, axillary and brachial arteries, it is limited in its ability to assess other scalp arteries.

This study has several limitations. As a single-centre retrospective analysis, findings may reflect local practice patterns and imaging protocols. Data were collected from routine care rather than a standardized protocol, resulting in variability in documentation, particularly around timing of glucocorticoid exposure and symptom onset. Some arteries, including the internal carotid and vertebral, were infrequently positive, leading to wide CIs and uncertain estimates. Additionally, our study only evaluated a predefined set of cranial vessels and did not assess others like the middle meningeal artery, facial artery or optic nerve sheath. Most imaging was performed with a small field of view specifically targeted to the ipsilateral superficial temporal artery which yielded the highest-resolution images and may exaggerate the sensitivity of temporal artery involvement compared with other vessels. Additionally, many of the initial scans did not include dedicated orbital or maxillary artery sequence, potentially leading to under-detection of abnormalities in these vessels. Finally, we saw that there was a negative relationship between glucocorticoid exposure and imaging/vessel positivity. This is consistent with previous data showing decreases in sensitivity in the first 24–96 h but shows an ongoing decreased sensitivity with a 5% relative decrease in the odds of positive imaging per day of glucocorticoid exposure out to 30 days [24]. Given that many cvMRIs were performed within weeks, rather than days of the commencement of glucocorticoids this was likely an important driver of the middling associations between symptom-vessel correlates. As clinicians were not blinded to cvMRI results, diagnostic bias is possible and we used a standard of the final diagnosis assigned after at least 6 months of follow-up to allow for confirmation over time.

These findings reaffirm the diagnostic value of cvMRI in GCA, highlighting utility alongside TA-US in diagnostically uncertain or atypical cases. Future studies should explore standardized imaging with shorter periods of glucocorticoid exposure to validate these associations in larger cohorts and further delineate the role of each imaging modality in the diagnosis of GCA.

## Supplementary Material

rkag060_Supplementary_Data

## Data Availability

The data underlying this article will be shared on reasonable request to the corresponding author.
