# Supplementary material for: Correlation of vascular enhancement on MRI and clinical manifestations in patients with GCA
Source: Rheumatol Adv Pract. 2026 Jul 3;10(3):rkag060. doi: 10.1093/rap/rkag060 (PMC13344854; doi:10.1093/rap/rkag060)
Supplement: rkag060_Supplementary_Data [file rkag060_supplementary_data.docx]

Supplementary material

SUPPLEMENTARY TABLE S1: Frequency and percent of vessel findings by symptoms of GCA in those with positive cranial vessel wall magnetic resonance imaging.

| **Presenting Symptoms** | **Temporal** | **Maxillary** | **Ophthalmic** | **Internal Carotid** | **Occipital** | **Vertebral** |
| --- | --- | --- | --- | --- | --- | --- |
| Vessel positive | 93 (60.78%) | 35 (22.88%) | 56 (36.6%) | 9 (5.88%) | 68 (44.44%) | 4 (2.61%) |
| Any Headache | 66 (55.93%) | 22 (18.64%) | 40 (33.9%) | 5 (4.24%) | 49 (41.53%) | 2 (1.69%) |
| Typical headache | 27 (71.05%) | 6 (15.79%) | 15 (39.47%) | 4 (10.53%) | 20 (52.63%) | 1 (2.63%) |
| Scalp Tenderness | 39 (59.09%) | 13 (19.7%) | 22 (33.33%) | 5 (7.58%) | 29 (43.94%) | 2 (3.03%) |
| Temporal Artery Tenderness | 32 (56.14%) | 13 (22.81%) | 21 (36.84%) | 2 (3.51%) | 25 (43.86%) | 0 (0%) |
| Jaw Claudication | 47 (79.66%) | 23 (38.98%) | 34 (57.63%) | 5 (8.47%) | 36 (61.02%) | 2 (3.39%) |
| Any vision changes | 59 (65.56%) | 26 (28.89%) | 39 (43.33%) | 7 (7.78%) | 46 (51.11%) | 4 (4.44%) |
| Vision loss | 29 (78.38%) | 13 (35.14%) | 21 (56.76%) | 1 (2.7%) | 24 (64.86%) | 1 (2.7%) |

ALT TEXT: Table lists associations between seven arteries and key GCA symptoms, including vision loss, scalp tenderness, jaw claudication, and headache.

SUPPLEMENTARY TABLE S2: Correlation between symptoms and side of vessel positivity in individuals with cvMRI-positive GCA (n=103). Individuals with bilateral symptoms and/or vessel abnormalities were counted on each side.

| Symptom | Artery | Side | Symptom present | Symptoms ipsilateral to abnormal vessel | Symptoms contralateral to abnormal vessel | Symptoms without vessel abnormality |
| --- | --- | --- | --- | --- | --- | --- |
| Typical headache | Temporal | Right | 44 | 22 | 20 | 2 |
|  |  | Left | 45 | 21 | 23 | 1 |
| Temporal artery tenderness | Temporal | Right | 20 | 11 | 8 | 1 |
|  |  | Left | 21 | 9 | 12 | 0 |
| Jaw claudication | Maxillary | Right | 28 | 6 | 6 | 16 |
|  |  | Left | 30 | 7 | 7 | 16 |
| Vision changes and/or vision loss | Ophthalmic | Right | 39 | 13 | 15 | 11 |
|  |  | Left | 41 | 16 | 14 | 11 |
| Vision Loss | Ophthalmic | Right | 13 | 5 | 6 | 2 |
|  |  | Left | 13 | 6 | 5 | 2 |

ALT TEXT: Table showing symptom, artery, and laterality (right/left), indicating whether symptoms occurred ipsilateral, contralateral, or without corresponding vessel abnormalities.
